# EHMTI-0196. Tension type headache – the correlation between pain intensity, depressive symptoms and impairment of daily functions

**DOI:** 10.1186/1129-2377-15-S1-C7

**Published:** 2014-09-18

**Authors:** M Bosnar Puretic, A Lovrencic-Huzjan, V Vukovic Cvetkovic, V Basic Kes

**Affiliations:** 1 University Department of Neurology, University Hospital Center "Sestre Milosrdnice", Zagreb, Croatia

## Introduction

Tension type headache is the most common type of headache. Although it is not a serious medical condition, in the general population it is the main cause of analgesics use.

## Aim

The aim of this study was to estimate intensity and other qualities of pain as well as the influence of pain on daily functions, the presence of depressive symptoms and their correlation with pain intensity in patients with tension type headache.

## Patients and methods

It was a prospective study performed at the University Department of Neurology, Sestre milosrdnice University Hospital Centre, Zagreb. Patients with tension type headache older than 18y and 30 healthy volunteers were included. Exclusion criteria were: patients younger than 18y, serious physical or mental illness and use of prophylactic therapy for headaches. The intensity and quality of pain were estimated by visual analog scale (VAS),McGill questionnaire,Brief Pain Inventory (BPI) and presence of depressive symptoms by Beck depression inventory (BDI-II). Descriptive statistical methods and regression analysis were used in statistical analysis.

## Results

The pain intensity in patients with tension type headache was according VAS 51,7+11,86/100mm, McGill questionnaire 15,13+5,06 points and according BPI 1,9+0,86 points. The disability level according BPI was 1,84+1,33 points. The presence of depressive symptoms was 8,76+4,57 points, significantly higher than in the control group (6,26+3,57). The regression analysis has not shown any correlation between intensity of pain and presence of depressive symptoms.

## Conclusion

The tension type headache is the chronic pain condition, probably the consequence and not the reason of depressive symptoms.

No conflict of interest.

